# Effects of alfalfa (*Medicago sativa* L.) supplementation in the diet on the growth, small intestinal histomorphology, and digestibility of hybrid ducks

**DOI:** 10.14202/vetworld.2021.2719-2726

**Published:** 2021-10-24

**Authors:** Bambang Suwignyo, Eprilia Aristia Rini, Muhammad Khoerul Fadli, Bambang Ariyadi

**Affiliations:** 1 Department of Animal Nutrition and Feed Science, Faculty of Animal Science, Universitas Gadjah Mada, Yogyakarta 55281, Indonesia; 2 Department of Poultry Production, Faculty of Animal Science, Universitas Gadjah Mada, Yogyakarta 55281, Indonesia.

**Keywords:** alfalfa, digestibility, histomorphology, hybrid ducks, performance

## Abstract

**Background and Aim:**

Feed plays the most important role in supporting livestock productivity. There is a need for studies on the nutrient levels in feed absorbed by the body of livestock. The aim of this study was to investigate the effects of alfalfa (*Medicago sativa* L.) supplementation in feed on growth, small intestinal histomorphology, and digestibility in hybrid ducks.

**Materials and Methods:**

The study was conducted *in vivo* using 75 hybrid ducks, with three treatments and five replications. Each replication consisted of five ducks. The treatments were: T0=basal ration without any alfalfa supplementation; T1=basal ration+3% fresh alfalfa supplementation; and T2=basal ration+6% fresh alfalfa supplementation. Fresh alfalfa was quantified on the basis of dry matter content. Drinking water was provided *ad libitum*. The observed variables were growth performance, measured in terms of feed consumption, body weight gain, and feed conversion ratio; duodenum histomorphology, measured in terms of villus height, villus width, and crypt depth; digestibility, measured in terms of dry matter digestibility; and organic matter, crude protein, and crude fiber concentrations. The data were analyzed using variance analysis with a completely randomized design of one-way pattern with Statistical Product for Service Solution application of version 22. The data with significant differences were further analyzed using Duncan’s new multiple range rest.

**Results:**

The results of the study showed that 3% alfalfa supplementation increased feed consumption and body weight gain of the hybrid ducks at 35 days of age compared with 0% and 6% supplementation. Furthermore, 3% alfalfa supplementation presented the best result in terms of villus height and duodenal crypt depth. Meanwhile, 6% alfalfa supplementation decreased nutrient digestibility in the ducks.

**Conclusion:**

These findings show that supplementation of feed with fresh alfalfa could have a significant effect on hybrid ducks in terms of growth performance, small intestinal histomorphology, and digestibility.

## Introduction

Livestock productivity is a major factor influencing the livestock industry. Livestock productivity can be improved by supplying nutritious feed and maintaining good management practices and an appropriate living environment for the livestock [1]. Feed plays the most important role in supporting livestock productivity and should be provided in an appropriately digestible condition and should contain nutrients at concentrations appropriate for the growing phase of the livestock for optimal productivity. Feed must contain nutrients that are in accordance with the needs of the poultry according to species, breed, and age [2].

Alfalfa is a forage containing essential nutrients such as proteins and crude fibers (CF) [3]. In addition, alfalfa contains beta-carotene, essential amino acids, and several secondary metabolites (flavonoids and saponins) and thus functions as an antioxidant [4]. Alfalfa is currently widely used as a feed for ruminants because ruminants can digest fiber and utilize it appropriately. Although poultry can digest fiber only in limited amounts, ducks have a higher tolerance to fibrous feed than other birds [5,6]. Thus, fiber supplementation in duck feed may improve the performance of ducks and decrease the cholesterol content in their meat and eggs [7,8].

Broiler chickens are only able to consume a maximum of 5% fiber in their feed [9], while ducks are able to digest up to 6% and 9% fiber without an increase in consumption [10]; 3%, 6%, and 9% fiber had no impact on the growth of ducks in the grower period [11]. Alfalfa is fibrous but has the potential to be used as feed for poultry because of its complete nutritional content, including essential amino acids [12], but this needs to be demonstrated. In the previous studies, supplementation of 6% alfalfa forage in duck feed was tested, performance was found to be improved in several aspects [6,13].

The presence of alfalfa in duck feed is expected to trigger peristaltic movements in the duck digestive tract, thereby facilitating the digestive process. This, in turn, would improve the absorption of nutrients in the small intestine, thereby increasing livestock productivity. Therefore, in this study, we aimed to further study the effects of alfalfa supplementation in the feed of hybrid ducks.

## Materials and Methods

### Ethical approval

The design and procedure of the study were approved by the Research Ethical Commission of the Faculty of the Veterinary Medicine UGM, Yogyakarta (No.:0021/EC-FKH/Eks/2019).

### Study period and location

The study was conducted from February 2019 to May 2019 in several locations: The Bird Cage of the Nutrient Biochemistry Laboratory of the Faculty of Animal Science of Universitas Gadjah Mada (UGM), Yogyakarta, Indonesia for the *in vivo* test of hybrid ducks, the Nutrient Biochemistry Laboratory of the Faculty of Animal Science of UGM for the proximate analysis of the excretes, and the Histology Laboratory of the Faculty of Animal Science of UGM for the histomorphological test of the small intestine of the hybrid ducks.

### Experimental design and management

We used 75 1-day-old hybrid ducks (Peking×Khaki Campbell), and the ducks were raised up to 35 days of age in 15 battery coops (125 cm×125 cm×50 cm). A Camry digital scale was used to weigh the feed (sensitivity 0.01) (5 kg) (PT. Insoclay Acidatama Indonesia, Tangerang, Indonesia). Portable digital scales were used for weighing ducks (sensitivity 0.1) (50 kg) (PT. Rohartindo Nusantara Luas, Tangerang, Indonesia). We used five feeding (capacity 1 kg) and drinking water containers (5 L) (PT. Medion Ardhika Bhakti, Bandung, Indonesia). The ducks were fed twice a day in the morning and evening; drinking water was provided *ad libitum*.

Alfalfa used for supplementation was quantified on the basis of dry matter, but it was provided in a fresh form. It was cut directly in the field before flowering, weighed, and cut into small pieces. The alfalfa pieces were provided to the ducks in a predetermined proportion.

The ducks (n=75) were assigned to three treatment groups with five replications. Each replication consisted of five ducks. The study used a completely randomized design. The treatments included the following: T0=basal ration without any alfalfa supplementation; T1=basal ration+3% fresh alfalfa supplementation; and T2=basal ration+6%fresh alfalfa supplementation. The basal ration used was a commercial product (BR-1;PT.JapfaComfeed Indonesia Tbk, Sragen, Indonesia) with 23% crude protein (CP), 21.5% ether extract, 5% CF, 7% ash, 0.8-1.1% calcium (Ca), and 0.5% phosphorous (P). The rations were prepared based on the need of the ducks according to the Indonesian National Standard (SNI) recommendations [14]. The materials and the ration contents are summarized in Table-1.

**Table-1:** Nutritional content of the hybrid duck diets.

Feed material	Treatment groups		

	T0	T1	T2
Commercial feed (%)	100	97	94
Alfalfa (%)	0	3	6
Total (%)	100	100	100
Nutrient content			
Metabolizable energy (kcal/kg)	3.000	2.958	2.917
Crude protein (%)	21.00	20.08	21.15
Crude fiber (%)	5.40	5.84	6.29
Crude fat (%)	3.80	3.99	4.17
Ca (%)	1.00	1.02	1.04
Available *P* (%)	0.27	0.26	0.26
Lysine (%)	1.07	1.04	1.01
Methionine (%)	0.49	0.48	0.46

T0=Commercial feed without fresh alfalfa supplementation, T1=Commercial feed+supplementation of 3% fresh alfalfa, T2=Commercial feed+supplementation of 6% fresh alfalfa

### Sampling and measurements

The parameters observed in the study were growth performance, measured in terms of feed consumption, body weight gain, and feed conversion ratio; histomorphology, measured in terms of villus height, villus width, and crypt depth; and digestibility, measured in terms of dry matter digestibility, feed consumption, and efficiency. The parameters were measured weekly. Feed wastage was recorded daily, and the data were used in the calculation of feed consumption.

Fifteen ducks (one per replication) were slaughtered and sampled at 35 days feeding trial. The small intestinal segments prepared for histological analysis were part of the duodenum with its folding wedge forming parallel loops. The sample of fresh small intestine was cut into 2-cm pieces, fixed in 10% buffered formalin, soaked for 24-48 h, and then prepared for histological analysis. The samples were prepared quickly by dehydrating them (soaking in a gradual series of alcohol-70%, 80%, 90%, and 100%). The small intestine samples were then cleared by soaking them in xylol and then embedded in paraffin. The samples were sectioned into 5-μm pieces and stained with hematoxylin and eosin. Finally, the samples were examined under a light microscope. The villus height, crypt depth, and villus depth were determined as reported by Sadeghi *et al*. [15]. The sections were observed at 4× magnification under a microscope with Optilab Viewer 2.2 (PT. Miconos Transdata Nusantara, Yogyakarta, Indonesia) connected to a laptop monitor. After analyzing the morphology of the small intestine, photographs of all histological preparations were taken for further measurements. The resulting photographs of the histomorphological samples were measured using Raster Image (PT. Miconos Transdata Nusantara, Yogyakarta, Indonesia) to obtain the villus height, villus width, and crypt depth.

The digestibility of the organic matter, dry matter, CP, and CF was measured using the total collection method. Excreta was collected using trays that were labeled according to treatment. The excreta was collected for 7 days when the ducks were 5 weeks of age. On day 1, the ducks were fasted to empty their digestive tract and were provided only drinking water. On days 2-7, the ducks were fed rations supplemented with 0% alfalfa (control), 3% fresh alfalfa, or 6% fresh alfalfa. The collected excreta samples were sprayed with 0.1 N HCl and then weighed to obtain the fresh weight and dry weight after drying. The total sample was weighed and maintained in a container specified for each treatment. The collected excreta samples were cleaned by removing any contaminating feathers and feed and then dried by aerating for 3 days without any direct exposure to sunlight. Subsequently, proximate analysis was performed to determine the digestibility of organic matter, dry matter, CF, and CP. Each experiment was carried out using 5 g of sample, with a total of 20 g/treatment for the five replications.

The proximate analysis was performed according to the Association of Official Analytical Chemist method [16]. The proximate analysis of dry matter, organic matter, CF, and CP was performed.

The dry matter analysis began by washing a silica disk and drying it in an oven for 1 h at 105-110°C, and then cooling in a desiccator for 15 min and weighing (X g). One gram of sample (Y g) was added to the silica disk and placed in an oven for 4-6 h at 105-110°C. The sample was then cooled in a desiccator for 15 min; after which, it was weighed (Z g). Drying was repeated until a constant sample weight was obtained (maximum difference 0.1 mg). Dry matter digestibility was determined as reported by Tilman *et al*. [17]:


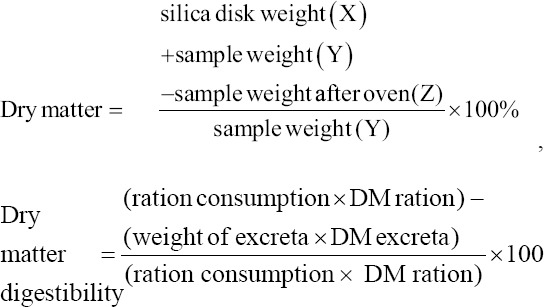


The ash content analysis began by cleaning the crucible and then drying it in an oven at 105-110°C for 1 h and cooling it in a desiccator for 15 min. The crucible was then weighed (X g); thereafter, the weighed sample (Y g) was added to the crucible. The sample was placed in an electric furnace at 400-600°C for 4-6 h. After turning off the electric furnace, the sample was cooled to120°C, moved to the desiccator for 15 min, and weighed (Z g). Organic matter digestibility was determined as reported by Tilman *et al*. [17]:


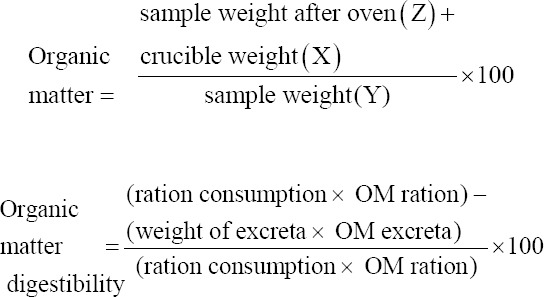


CF analysis was carried out by cleaning all glassware and drying it in an oven at 105-110°C. Whatman 41 filter paper was dried in an oven at 105-110°C for 1 h and then weighed. One gram of sample was weighed and added into a 250-mL glass beaker. Subsequently, 50 mL of 0.3 N H_2_SO_4_ was heated to boiling for 30 min; 25 mL of 1.5 N H_2_SO_4_ was added to the sample, which was then heated again to boiling for 30 min. The solution was filtered using filter paper attached to a Buchner funnel on a vacuum pump. The precipitate was then washed successively with 50 mL of hot distilled water, dissolved in 50 mL of 0.3 NH_2_SO_4_, 50 mL of hot water, and finally with 25 mL of acetone/N-hexane, and then allowed to dry. After drying, the filter paper and its contents were placed in a crucible and then dried in an oven at 105-110°C for 6-12 h. The sample was cooled in a desiccator for 15 min and weighed. CF digestibility was determined as described by Tilman *et al*. [17]:


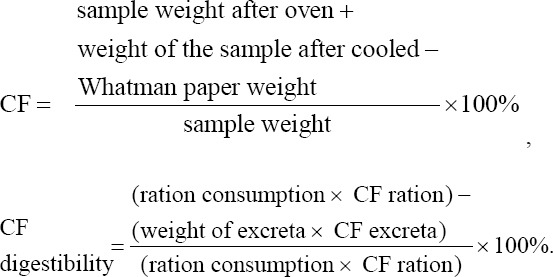


Analysis of CP content was carried out by adding approximately 0.3 g of sample, 0.3 g of catalyst selenium reagent mixture, and 10 or 15 mL of H_2_SO_4_ to a digestion flask or Kjeldahl flask. The flask was heated in the fume hood until the color of the solution turned clear green. We added 20 mL of 4% H_3_BO_3_ solution into a 250-mL Erlenmeyer flask, to which two drops of the MR+MB mixture indicator were added. The digested sample was added into a distillation flask, to which 50 mL of distilled water and 40 mL of 45% NaOH were added. The distillation process was carried out until the solution changed from purple to green. The solution was then titrated with 0.1 N HCl until the color changed from green to purple. CP digestibility was determined as described by Tilman *et al*. [17]:


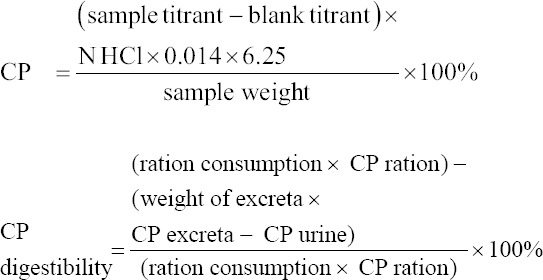


CP excreta= [(total excreta× (CP excreta× 70%); uric acid nitrogen was assumed to be 30% of nitrogen excreta [18].

### Statistical analysis

All data, including growth performance, small intestinal histomorphology, and digestibility data, were analyzed using a one-way completely randomized design with SPSS version 22 (SPSS Armonk, New York, USA). The data with significant differences were further analyzed using Duncan’s new multiple range test. The data variability is expressed as standard error of the means, and results with p<0.05 were considered to be significant.

## Results

### Growth performance

The growth performance of the hybrid ducks after alfalfa supplementation is shown in Table-2, which shows that alfalfa supplementation had a significant effect on feed intake and body weight gain of hybrid ducks (p<0.05). The feed intake and body weight gain of the hybrid ducks increased on 3% fresh alfalfa supplementation. Ration with alfalfa supplementation of 6% and ration without alfalfa supplementation decreased the body weight gain of the hybrid ducks. The alfalfa supplementation in the feed (Table-2) did not have any significant effect on the feed conversion ratio.

**Table-2:** The impact of the alfalfa supplementation on the production performance of the hybrid ducks.

Variables	Alfalfa supplementation levels (%)		

	0	3	6
FI (g/ekor)	2517.05±35.88^b^	2711.10±35.18^a^	2492±15.57^b^
BWG (g/ekor)	978.70±21.02^b^	1064.36±31.64^a^	946.67±15.30^b^
FCR^ns^	2.57±0.16	2.55±0.24	2.63±0.19

a, b=The numbers with different superscripts in the same row were indicative of significant difference (p<0.05); ns=Insignificantly different. FI=Feed intake; BWG=Body weight gain; FCR=Feed conversion ratio

### Small intestinal histomorphology

**Figure-1:**
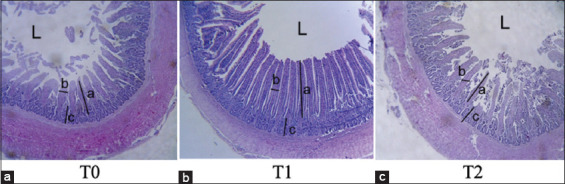
The resulting histomorphology of the duodenum of the small intestine of the hybrid ducks after treatments of alfalfa supplementation. a, villus height; b, villus width; c, crypt depth; and L, small intestinal lumen at 4× microscopic magnification. T0=commercial feed without fresh alfalfa supplementation, T1=commercial feed+supplementation of 3% fresh alfalfa, T2=commercial feed+supplementation of 6% fresh alfalfa.

Based on the results in Table-3, alfalfa supplementation had a significant effect on the villus height, villus width, and crypt depth of the duodenum of the hybrid ducks (p<0.05). Fresh alfalfa supplementation at 3% presented the highest villus height (Figure-1). There was no significant difference in the villus width between 3% and 6% fresh alfalfa supplementation and the control. The results of the study showed that 3% fresh alfalfa supplementation resulted in the deepest crypt depth, which might be related to the feed intake. Table-3 shows that 3% fresh alfalfa supplementation resulted in the highest feed intake.

**Table-3:** The impact of alfalfa supplementation in the ration on the histomorphology of the duodenum of the hybrid ducks.

Parameters	Alfalfa supplementation level (%)		

	0	3	6
Villus height (μm)	746.14±14.34^b^	828.88±12.95^a^	711.33±10.69^c^
Villus width (μm)	83.23±1.72^a^	76.70±3.92^ab^	72.84±1.94^b^
Crypt depth (μm)	185.90±1.35^b^	245.73±5.63^a^	134.78±4.23^c^

a, b=Different superscripts in the same row are indicative of significant differences (p<0.05)

### Nutrient digestibility

The results summarized in Table-4 show that all parameters of nutrient digestibility under 3% fresh alfalfa supplementation did not significantly differ (p<0.05) from those under 6% and 0% fresh alfalfa supplementation. The alfalfa supplementation decreased dry matter, organic matter, CP, and CF digestibility in the hybrid ducks.

**Table-4:** Effect of alfalfa supplementation on nutrient digestibility of hybrid duck.

Parameters	Alfalfa supplementation level (%)		

	0	3	6
Dry matter digestibility	66.60±4.69^a^	65.46±2.26^ab^	60.70±3.52^b^
Organic matter digestibility	73.50±3.89^a^	72.16±1.74^ab^	68.19±2.88^b^
Crude protein digestibility	68.57±4.42^a^	65.95±1.8^ab^	59.71±7.02^b^
Crude fiber digestibility	77.80±4.96^a^	65.95±11.97^ab^	60.18±9.84^b^

a, b=Different superscripts in the same row are indicative of significant differences (p<0.05)

## Discussion

Growth performance is an important parameter used to determine whether a treatment will have a negative or positive effect on livestock. In the present study, 3% fresh alfalfa supplementation increased growth performance, but 6% fresh alfalfa supplementation decreased growth performance. The increase in feed intake under 3% fresh alfalfa supplementation correlated with the proportion of CF contained in alfalfa. The proportion of CF under 3% fresh alfalfa supplementation could still be tolerated by the ducks, as indicated by the increase in the feed intake with 3% fresh alfalfa supplementation. According to Rini *et al*. [19], ducks could tolerate higher CF compared with other poultry. Ducks have been reported to tolerate fiber content up to 10% [6].

However, the feed intake decreased under 6% fresh alfalfa supplementation because the alfalfa content in the ration was higher than the proportion of CF contained in the ration. According to Prawitasari *et al*. [20], a high CF concentration in the feed results in longer nutrient digestion and, consequently, decreases the energy value. Given that high-CF feed is bulky in nature, the high CF concentration caused the ducks to feel sated, thereby decreasing feed intake. The decrease in feed intake had a significant effect on the decrease in body weight gain.

According to Jiang *et al*. [11], 3%, 6%, and 9% fresh alfalfa supplementation did not increase feed consumption of Chinese ducks. Ashshofi *et al*. [21] suggested that the mean consumption of commercial feed by male hybrid ducks for 4 weeks was 2800 g/duck. Sigit and Sasongko [22] pointed out that the feed intake of male Magelang ducks in the raising period of 5 weeks with commercial feed was in the range of 2433-2485 g. Christian *et al*. [23] suggested that the feed consumption of Mojosari ducks of 5 weeks of age with basil flour supplementation was in the range of 2298-2602 g.

The body weight gain of ducks is influenced by feed intake, and the increase in feed intake would certainly be followed by body weight gain if there are no physiological disorders in the digestive tract of the ducks. According to Afsharmanesh and Mehdipour [24], *ad libitum* or restricted feeding can increase body weight gain and cause an increase in feed intake to growth rate. Nassar *et al*. [25] reported that providing a high-fiber diet increased chicken body weight gain, followed by an increase in feed intake.

The results of the variance analysis showed that 6% fresh alfalfa supplementation decreased the body weight gain of the hybrid ducks. This may have been caused by the decrease in feed intake, or it might have occurred because of the digestive physiological process. Mateos *et al*. [26] concluded that the potential response to fiber supplementation might depend on the source and level of dietary fiber supplementation, the properties of the diet, the physiological status, and the health of the ducks. The dietary fiber levels might cause differences in the gastrointestinal tract transit rate, pH value, and volatile fatty acid production in poultry [27]. Consequently, the dietary fiber levels might affect the voluntary feed intake, organ size, gastrointestinal tract motility, enzyme production, nutrient digestibility, microbial growth, and growth performance. According toAshshofi *et al*. [21], the mean body weight gain of the male hybrid ducks fed a commercial feed for 4 weeks was 900 g/duck.

Alfalfa supplementation (Table-2) did not have a significant effect on feed conversion ratio. This indicated that the three treatments were similarly efficient. The difference in the performance of the ducks in the three treatments was reflected in the quantity of the consumed feed. The more the feed was consumed, the higher the body weight gained.

The results of feed conversion with alfalfa supplementation in the present study were better than those reported by Purba and Prasetya [10]. The mean feed conversion results of the EPMp ducks, that is, ducks obtained by crossbreeding Manuila, Peking, and white Mojosari ducks, provided a feed containing 6% and 9% CF in the raising period of 12 weeks at 4.95 and 4.94, respectively. Ashshofi *et al*. [21] showed that the male hybrid ducks provided commercial feed until 5 weeks of age had a mean feed conversion 3.1 times higher than that in the present study.

The villus of the small intestine absorbs the majority of nutrients, and its height is indicative of the absorption capacity of the small intestinal mucosa. A higher intestinal villus height results in an increased contact surface width between the enterocytes and nutrients, resulting in improved absorption of the nutrients [28]. Thus, the villus height could influence the nutrient absorption capacity of the small intestine [29]. An increase in the villus height and width indicates efficient nutrient transportation in the body of the ducks. The smooth feed flow in the digestive tract is one of the influencing factors of the increase in villus width. The activity of the intestine might influence the increase in villus surface width to absorb nutrients [30]. The intestinal villus in birds can grow optimally if the need for nutrients in the growing period is met. The absorption of nutrients in the intestine could trigger the widening of the villus. An increase in the intestinal villus height indicates that the small intestine is working efficiently at digesting feed. Efficient intestinal absorption reflects a healthy intestinal condition, which can improve the ability to absorb nutrients [31].

Fresh alfalfa supplementation would increase the CF concentration in the feed. The CF contained in the feed could influence the development of the intestinal villus. Insufficient CF concentration might be a causal factor in the lack of height and width development of the intestinal villus. Consequently, the absorption of nutrients was not optimal. The supplementation of fiber in the feed could help the small intestine digest the feed and widen the villus [32]. Dietary fiber concentration influenced the morphological changes in the animal intestine, especially because of the changes in the surface width, including the height and the number of villi [33]. An increase in the surface area of the villi correlates with an increase in the rate of mucosal cell proliferation [34].

Table-2 shows that 3% fresh alfalfa supplementation resulted in the highest feed intake. Jiang *et al*. [11] suggested that feed intake influenced the villus height of the small intestine and crypt depth. An increase in feed intake triggered the development of the small intestine, which might be due to the influence of some fibrous components on the composition of the microorganisms in the digestive tract, especially the small intestine and the caecum, through fermentation, increasing the villus height and crypt depth in the bird’s small intestine. Fiber supplementation in the feed could stimulate the formation of volatile fatty acids, resulting from the fermentation by microorganisms in the small intestine and caecum. On the contrary, butyric acid could improve small intestine cell production, which may increase the crypt depth [35].

Alfalfa supplementation decreased the dry matter digestibility of the hybrid ducks. CF supplementation caused a decrease in feed intake as the CF component increased. Rompas *et al*. [36] reported that a large quantity of fiber components (lignin and silica) that were not digested resulted in low digestibility, and it was corroborated by the findings of Murray [37], suggesting that the large quantity of CF in the feed consumed by a duck caused a high feed movement rate in the digestive tract, resulting in a decreased working period of the digestive enzyme and decreased digestibility. Rompas *et al*. [36] reported that a large quantity of the digested dry matter correlated with the quantity of the absorbed nutrients.

Organic matter digestibility is influenced by the digestibility of other components. Mangisah *et al*. [38] reported that the digestibility of organic matter is influenced by the digestibility of organic matter components, including protein, carbohydrate (nitrogen-free extract and CF), and fat. The digestibility of organic matter is influenced by the high alfalfa nutrient concentration of the feed. The supplementation and change in feed nutrients increased the quality of the nutrients, ultimately increasing the role of alfalfa in the gastrointestinal tract organs of the ducks. Rompas *et al*. [36] suggested that the factor influencing the digestibility of organic matter is the nutrient concentration. The digestibility of organic matter would have a significant effect on the digestibility of CF, CP, and organic matter. The increase in the digestibility of dry matter would increase the digestibility of its organic matter.

The digestibility of CP depends on the protein concentration in the feed, quantity of protein in the digestive tract, environmental temperature, and physiological conditions of the livestock. The quantity of consumed CP influences the digestibility of the CP. The lower the CP contents in the feed, the lower the digestibility, and vice versa [20]. The increase in feed intake up to the optimal point would improve the digestibility performance of the ducks [39]. One of the steps to meet the digestion standards of amino acids is to decrease feed energy to ensure that amino acids could be more easily digested [40].

A feed with a high CF concentration resulted in a low intake of nutrients, resulting in decreased body weight. The digestibility of CF was affected by the CF concentration of the consumed feed and CF quantity. The digestibility of CF was influenced by several factors such as feed particle size, availability of microflora to digest feed in the small intestine, CF content, energy, protein content, and age of livestock [41].

Feed consumption was also influenced by the CF concentration of the feed. The higher the CF content, the lower the feed consumption because feed with a high fiber concentration is bulky in nature, limiting its consumption. These results are consistent with those of Pangestu *et al*. [42] and Suwignyo *et al*. [13]. CF has bulky properties and consists of cellulose, hemicellulose, and lignin that are difficult for ducks to digest. The bulkiness of the feed results in the saturation of the digestive tract, and therefore, the ducks would stop consuming the feed. Raninen *et al*. [27] reported that the concentration of CF would influence the duration of feed in the digestive tract, pH of the feed, and fatty acid content in the body of birds in flight. Maghfiroh *et al*. [41] described CF to be a type of carbohydrate consisting of cellulose, hemicellulose, and lignin, which could not be digested by birds and served only as bulky stomach fillers. Ducks would stop consuming feed when their need for energy has been met.

## Conclusion

It could be concluded that 3% fresh alfalfa supplementation increased the consumption and body weight gain of hybrid ducks. The best intestinal histomorphology was observed with 3% fresh alfalfa supplementation. We found that 6% fresh alfalfa supplementation decreased nutrient digestibility in hybrid ducks at 35 days of age. The optimal results were obtained with 3% fresh alfalfa supplementation.

## Authors’ Contributions

BS: Designed and guided the study and reviewed the manuscript. BS and EAR: Managed the study and wrote the manuscript. EAR and MKF: Collected and analyzed the samples. BA: Reviewed the manuscript. All authors read and approved the final manuscript.
